# Identification of genetic loci and candidate genes related to soybean flowering through genome wide association study

**DOI:** 10.1186/s12864-019-6324-7

**Published:** 2019-12-16

**Authors:** Minmin Li, Ying Liu, Yahan Tao, Chongjing Xu, Xin Li, Xiaoming Zhang, Yingpeng Han, Xue Yang, Jingzhe Sun, Wenbin Li, Dongmei Li, Xue Zhao, Lin Zhao

**Affiliations:** Key Laboratory of Soybean Biology of Ministry of Education, China (Key Laboratory of Biology and Genetics & Breeding for Soybean in Northeast China), Northeast Agricultural University, Harbin, China

**Keywords:** Genome wide association study, Candidate genes, Soybean growth periods, Genetic improvement

## Abstract

**Background:**

As a photoperiod-sensitive and self-pollinated species, the growth periods traits play important roles in the adaptability and yield of soybean. To examine the genetic architecture of soybean growth periods, we performed a genome-wide association study (GWAS) using a panel of 278 soybean accessions and 34,710 single nucleotide polymorphisms (SNPs) with minor allele frequencies (MAF) higher than 0.04 detected by the specific-locus amplified fragment sequencing (SLAF-seq) with a 6.14-fold average sequencing depth. GWAS was conducted by a compressed mixed linear model (CMLM) involving in both relative kinship and population structure.

**Results:**

GWAS revealed that 37 significant SNP peaks associated with soybean flowering time or other growth periods related traits including full bloom, beginning pod, full pod, beginning seed, and full seed in two or more environments at -log_10_(*P*) > 3.75 or -log_10_(*P*) > 4.44 were distributed on 14 chromosomes, including chromosome 1, 2, 3, 5, 6, 9, 11, 12, 13, 14, 15, 17, 18, 19. Fourteen SNPs were novel loci and 23 SNPs were located within known QTLs or 75 kb near the known SNPs. Five candidate genes (*Glyma.05G101800*, *Glyma.11G140100*, *Glyma.11G142900*, *Glyma.19G099700*, *Glyma.19G100900*) in a 90 kb genomic region of each side of four significant SNPs (Gm5_27111367, Gm11_10629613, Gm11_10950924, Gm19_34768458) based on the average LD decay were homologs of Arabidopsis flowering time genes of *AT5G48385.1*, *AT3G46510.1*, *AT5G59780.3*, *AT1G28050.1*, and *AT3G26790.1*. These genes encoding FRI (FRIGIDA), PUB13 (plant U-box 13), MYB59, CONSTANS, and FUS3 proteins respectively might play important roles in controlling soybean growth periods.

**Conclusions:**

This study identified putative SNP markers associated with soybean growth period traits, which could be used for the marker-assisted selection of soybean growth period traits. Furthermore, the possible candidate genes involved in the control of soybean flowering time were predicted.

## Background

Soybean (*Glycine max*) is a major crop of agronomic importance grown across a wide range of latitudes from 50°N to 35°S [1]. However, soybean varieties are limited to narrow latitudes due to the photoperiod sensitivity. The complex growth period traits are controlled by both internal and external factors, which make great effects on crop adaptability, biomass and economic yield [2]. As a typical photoperiod-sensitive short-day plant, soybean photoperiod is the main climatic factor that determines its growth periods and adaptability to different ecological zones. The genetic mechanisms of soybean flowering time and maturity were complex [3]. Previous studies identified at least 11 major-effect loci affecting flowering and maturity of soybean, which were designated as *E1* to *E10* [4–14], and the *J* locus for “long juvenile period” [15], which was important for soybean to adapt to high latitude environments. *E1*, *E2*, *E3*, *E4*, *E9* and *J* had been cloned or identified. Of these, *E1* encoding a nuclear-localized B3 domain-containing protein was induced by long days. *E2* encoded a homolog of *GIGANTEA* and controlled soybean flowering time by regulating *GmFT2a* [1]. *E3* and *E4* encoded phytochrome PHYA3 and PHYA2 proteins [7, 16]. *J* was the dominant functional allele of *GmELF3* [17]. In addition to these major loci, many minor-effect quantitative traits loci (QTLs) related to soybean flowering time and maturity had also been identified. To date, at least 104, 6, 5, and 5 QTLs associated with first flower, pod beginning, seed beginning, and seed fill had been reported in soybean (SoyBase, www.soobbase.org), respectively. Many other orthologs of Arabidopsis flowering genes such as *GmCOLs* [18], *GmSOC1* [19], and *GmCRY* [20] had also been identified. Taken together, these results showed a complex genetic basis of flowering and maturity in soybean.

Genome-wide association study (GWAS), based on linkage disequilibrium (LD), had emerged as a powerful tool for gene mapping in plants to take advantage of phenotypic variation and historical recombination in natural populations and overcome the limitations of biparental populations, resulting in higher QTL mapping resolution [21–23]. So far, the next-generation sequencing technologies such as genotyping by sequencing (GBS), restriction site-associated DNA sequencing (RAD-seq) and specific-locus amplified fragment sequencing (SLAF-seq) had been used to detect high-quality single nucleotide polymorphisms (SNPs) for GWAS in soybean [24–26]. The Illumina Infinium SoySNP50K BeadChip was used to genotype the population consisting of 309 early-maturing soybean germplasm resources, and ten candidate genes homologous to Arabidopsis flowering genes were identified near the peak SNPs associated with flowering time detected via GWAS [3]. Ninety-one soybean cultivars of maturity groups (MGs) 000-VIII were subjected to GWAS using Illumina SoySNP6K iSelectBeadChip, and 87 SNP loci associated with soybean flowering were identified [27]. Eight hundred and nine soybean cultivars were sequenced on Illumina HiSeq 2000 and 2500 sequencer, GWAS identified 245 significant genetic loci associated with 84 agronomic traits by single and multiple marker frequentist test (EMMAX), 95 of which interacted with other loci [28]. The recombinant inbred line (RIL) population were genotyped by RAD-seq in 2 year studies, the high-density soybean genetic map was constructed and 60 QTLs that influenced six yield-related and two quality traits were identified [29]. SLAF-seq technology had several obvious advantages, such as high throughput, high accuracy, low cost and short cycle, and this technology had been reported in haplotype mapping, genetic mapping, linkage mapping and polymorphism mapping. It could also provide important bases for molecular breeding, system evolution and germplasm resource identification. A total of 200 diverse soybean accessions with different resistance to SCN HG Type 2.5.7 were genotyped by SLAF-seq for GWAS, and the results revealed 13 SNPs associated with resistance to SCN HG Type 2.5.7, and 30 candidate genes underlying SCN resistance were identified [30]. In the present study, we performed GWAS for soybean growth period traits in the total of 278 soybean accessions genotyped by SLAF-seq and identified 37 significantly associated SNPs in two or more environments and five potential candidate genes regulating growth periods. Our studies provided an insight into the genetic architecture of soybean growth periods and the identified candidate markers and genes would be valuable for the marker-assisted selection of soybean.

## Results

### Phenotype statistics of 278 soybean germplasms

**Fig. 1:**
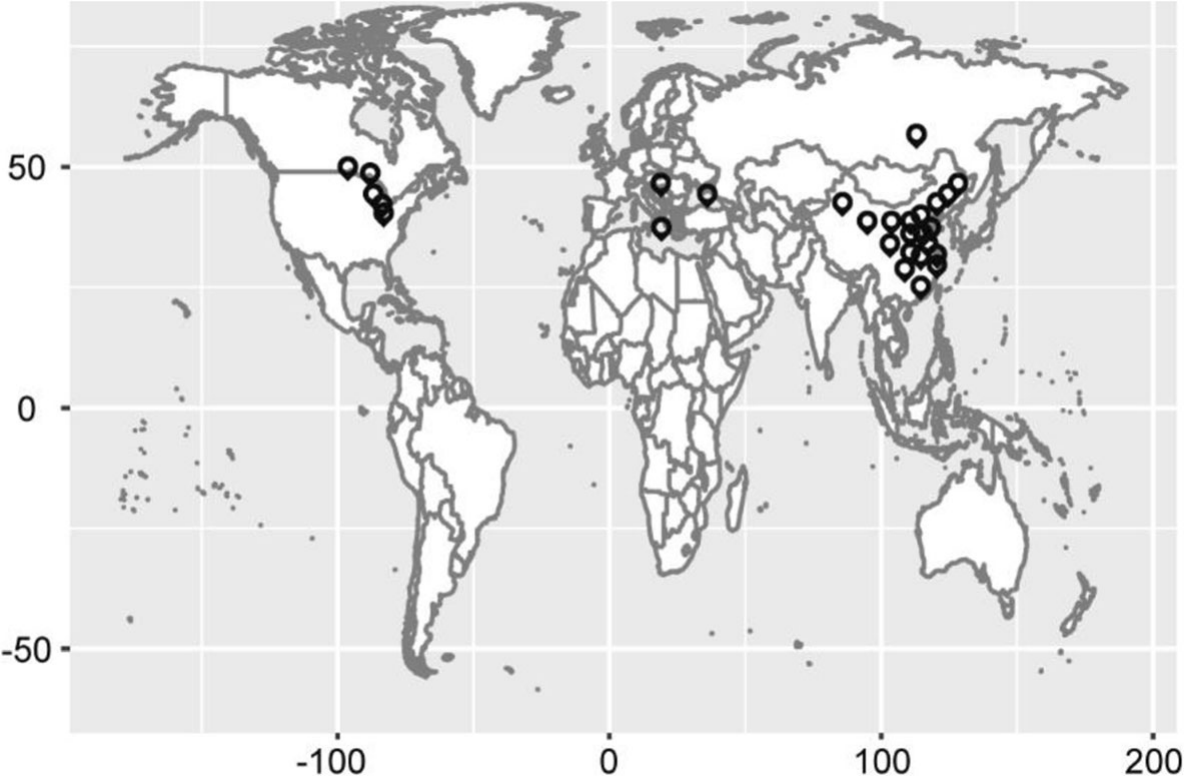
Geographical distribution of 278 soybean germplasm resources. The map was made by the completely free software R [31] version 3.6.1 (https://mirrors.tuna.tsinghua.edu.cn/CRAN/)

Field experiments were conducted in three different locations (Harbin, Changchun, Shenyang) in China for 2 years (2015 and 2016). The statistical analysis on the results of phenotype indicated that six growth period characteristics including flowering time, full bloom, beginning pod, full pod, beginning seed, and full seed of 278 soybean germplasms (Fig. 1, Additional file 1) showed abundant phenotypic variation (14.9~43.6%) (Additional file 2), and reflected their great potential of genetic improvement. After normalizing, the six growth period characters of 278 soybean germplasms above showed normal distributions without any significant skewness, which could be used for the subsequent statistical analysis (Additional file 10: Figure S1). Correlation analysis showed that there were high correlations between flowering time and full bloom (0.90~0.98), beginning pod (0.96~0.88), full pod (0.87~0.94), beginning seed (0.84~0.93), and full seed (0.83~0.90) (Additional file 11: Figure S2), implying that the flowering time and the other five growth periods in soybean might be controlled by the same genetic factors.

The results of ANOVA showed that the heritability of flowering time, full bloom, beginning pod, full pod, beginning seed, and full seed in soybean were quite high (94.7~96.2%) (Additional file 3), indicating that the growth periods traits were mainly significantly affected by genetic variability. Therefore, the probability of obtaining the off springs with excellent target traits was large by selecting them in the early generation of breeding using a strict criteria [32]. However, the flowering time, full bloom, beginning pod, full pod, beginning seed, and full seed in soybean were also affected by environmental factors such as geographical location and year, as well as environment-genotype interactions (*P* < 0.01) (Additional file 3), which made the majority of soybean bloom the earliest in Shenyang (lower latitude), whereas bloom the latest in Harbin (higher latitude) in the same year (Additional file 1, Additional file 2). Forty-one soybean germplasms flowering earlier (27.5~38.5 d) and 53 flowering later (58~113 d) with stable performance (Additional file 4) were screened by GGE biplot in six environments to avoid the impact of the environment, which could be considered for broadening the genetic basis for the improvement of soybean germplasms to produce greater super-parent effects.

### Linkage disequilibrium (LD), population structure and kinship analyses

The DNA sequencing data had been uploaded [33]. The dataset of 34,710 SNPs with MAF higher than 0.04 covering all 20 chromosomes was used to conduct GWAS (Additional file 5, Additional file 12: Figure S3). The largest number of SNPs was identified on chromosome 18 (2708 SNPs) followed by chromosome 15 (2515 SNPs), and the smallest of SNPs was found on chromosomes 11 (961 SNPs) and chromosomes 12 (1079 SNPs) (Additional file 6, Fig. 2). The highest marker density was detected on chromosome 15 (one SNP per 20.58 kb), and the smallest one was identified on chromosome 12 (one SNP per 37.15 kb), while the average marker density was approximately one SNP per 28.36 kb (Additional file 6). It was found that the average LD decay distance of the population was about 300 kb (r^2^ = 0.5) by 34,710 SNP markers for LD analysis (Fig. 3a). Previous studies had shown that the LD decay distance of soybean natural population was 250~375 kb [34], which was similar to the results of this study, indicating that the marker coverage obtained in this study was high enough for GWAS. The population structure of 278 soybean accessions obtained by principal component analysis of 34,710 SNPs reflected the subgroup structure (Fig. 3b and c), suggesting that geographic isolation was important for shaping genetic differentiation of soybean. The kinship matrix among 278 soybean accessions calculated based on 34,710 SNPs indicated a lower level of genetic relatedness among soybean individuals (Fig. 3d).

**Fig. 2:**
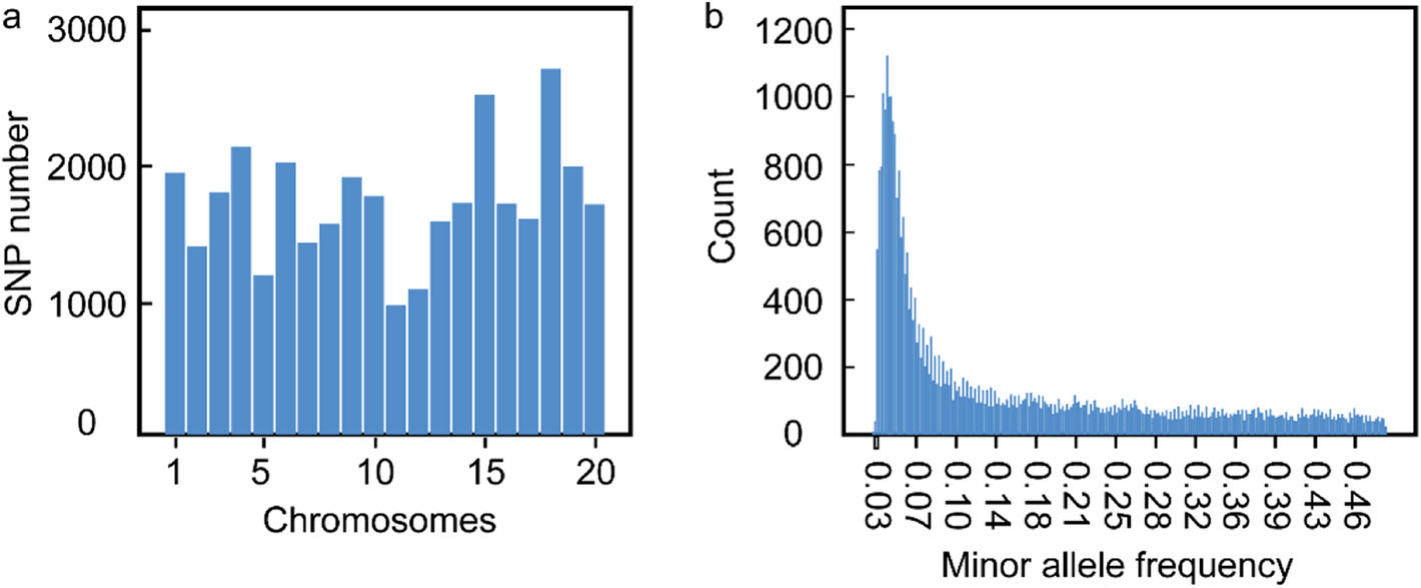
Single-nucleotide polymorphism for 278 soybean accessions. **a** Distribution of the SNP markers across 20 soybean chromosomes. **b** Minor allele frequency distribution of SNP alleles

**Fig. 3:**
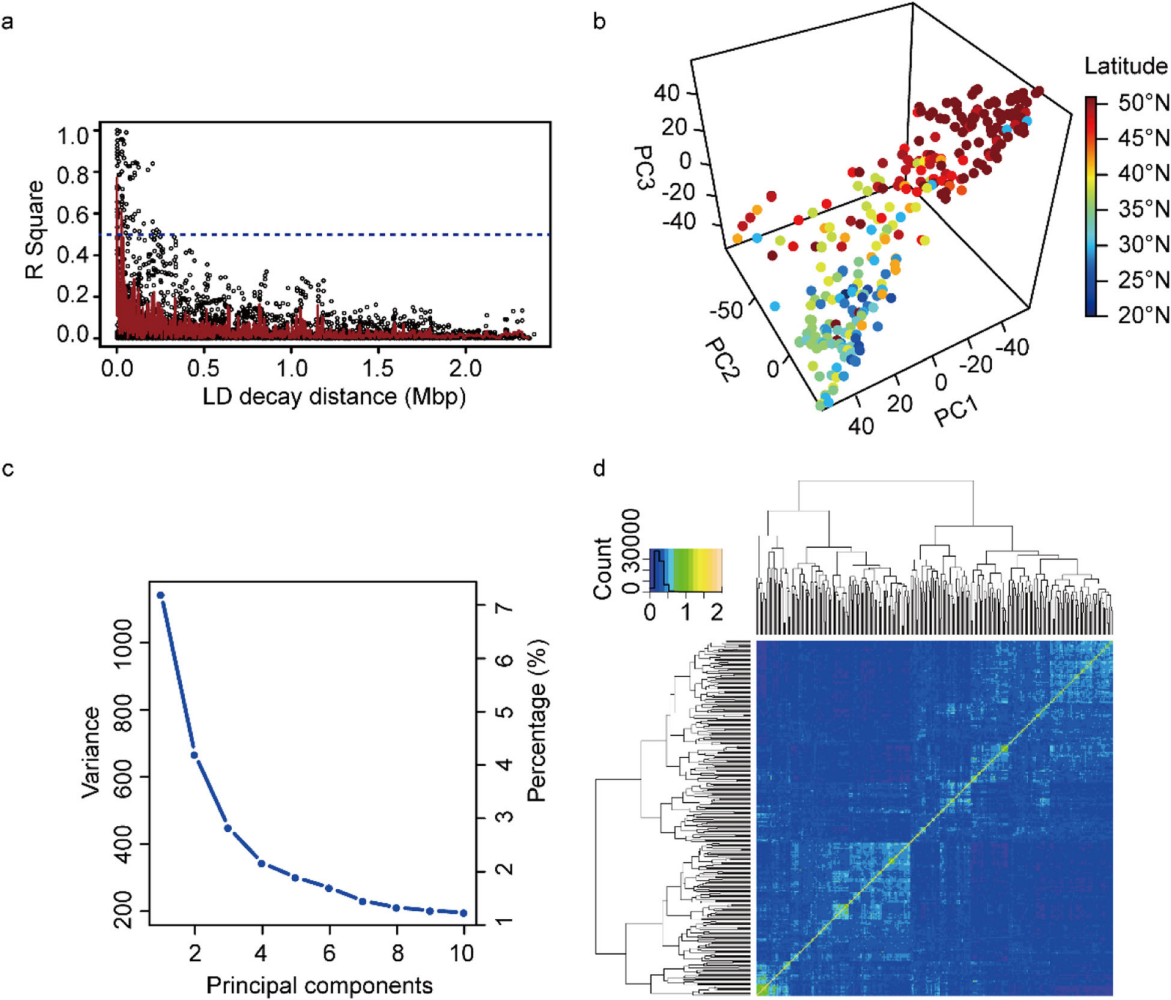
The linkage disequilibrium (LD), principal component and kinship analyses of soybean genetic data. **a** The estimated average linkage disequilibrium (LD) decay of soybean genome. The dashed line in blue indicated the position where r^2^ was 0.5. **b** The first three principal components of 34,710 SNPs used in the GWAS indicated little population structure among 278 tested accessions. **c** The population structure of the soybean germplasm collection reflected by principal components. **d** The heat map of the kinship matrix of the 278 soybean accessions calculated from the same 34,710 SNPs used in the GWAS, suggesting low levels of relatedness among 278 individuals

### Identification of genetic loci and candidate genes through GWAS

**Fig. 4:**
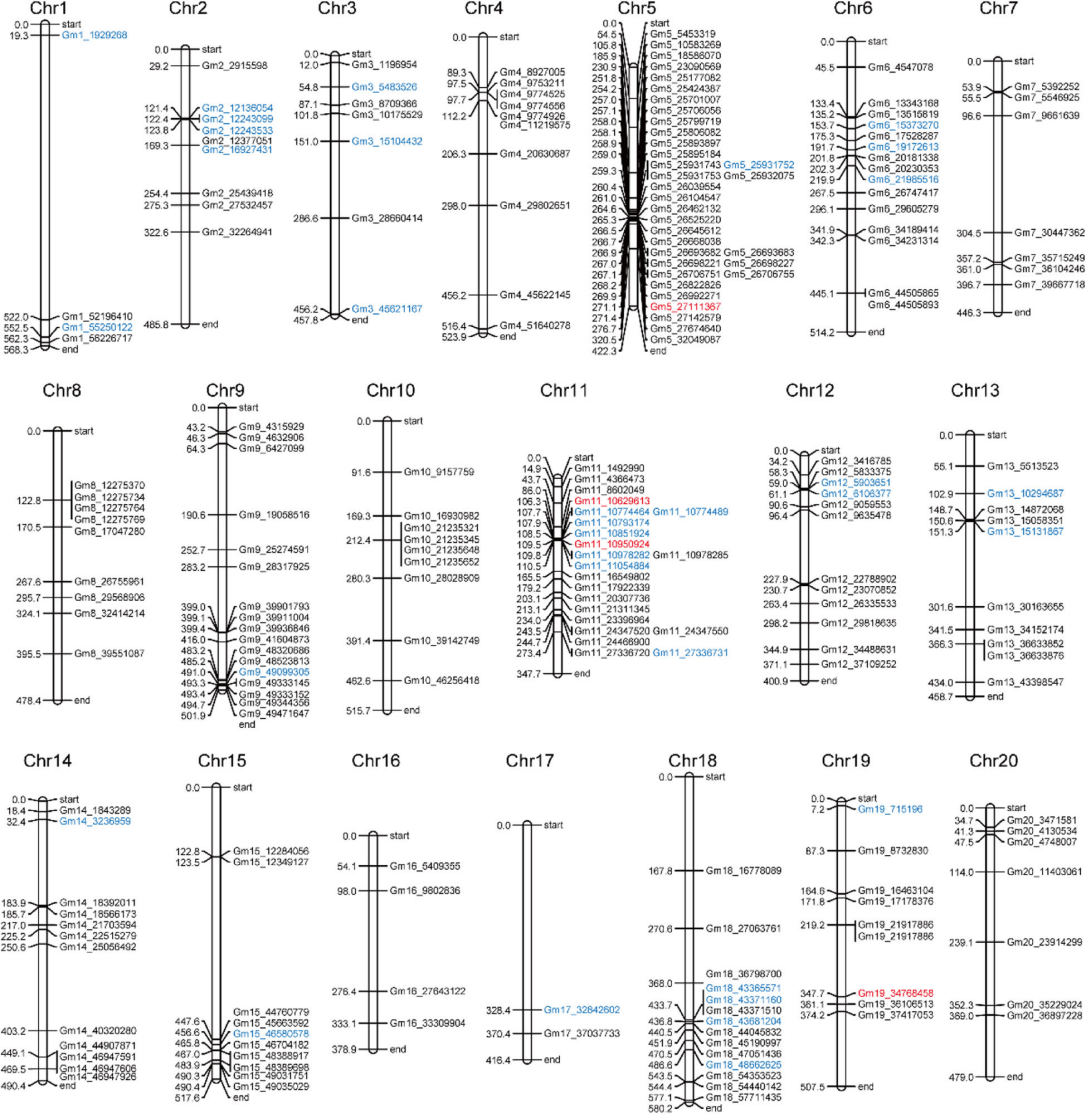
The positions of flowering time-related SNP loci on the chromosomes. The SNP loci associated with soybean flowering time and other growth periods in one or more environments were labeled black or blue, respectively. The soybean flowering candidate genes were then found in the linkage disequilibrium block of four SNP sites associated with soybean flowering found in multiple environments, which were marked red. The left number of each chromosome showed the relative in the genome, 1 = 100 kb

The CMLM-PCA + K statistical model considering the covariates composed of population structure and kinship matrix was used for GWAS to prevent false positivity [35]. The total of 223 SNP loci associated with flowering time, full bloom, beginning pod, full pod, beginning seed, and full seed in one or more environments were all considered to be candidate sites for flowering time in soybean, because the correlation analysis above demonstrated that these six growth period traits may be controlled by the same genetic factors (Fig. 4, Additional file 7, Additional file 8). Among them, 186 SNPs detected in one environment may be susceptible to environmental influences, 37 SNPs that could explain 17.41~21.95% phenotypic variation in two or more environments could be stably inherited in different environments, and it was considered that there would be key genes controlling flowering time nearby.

**Fig. 5:**
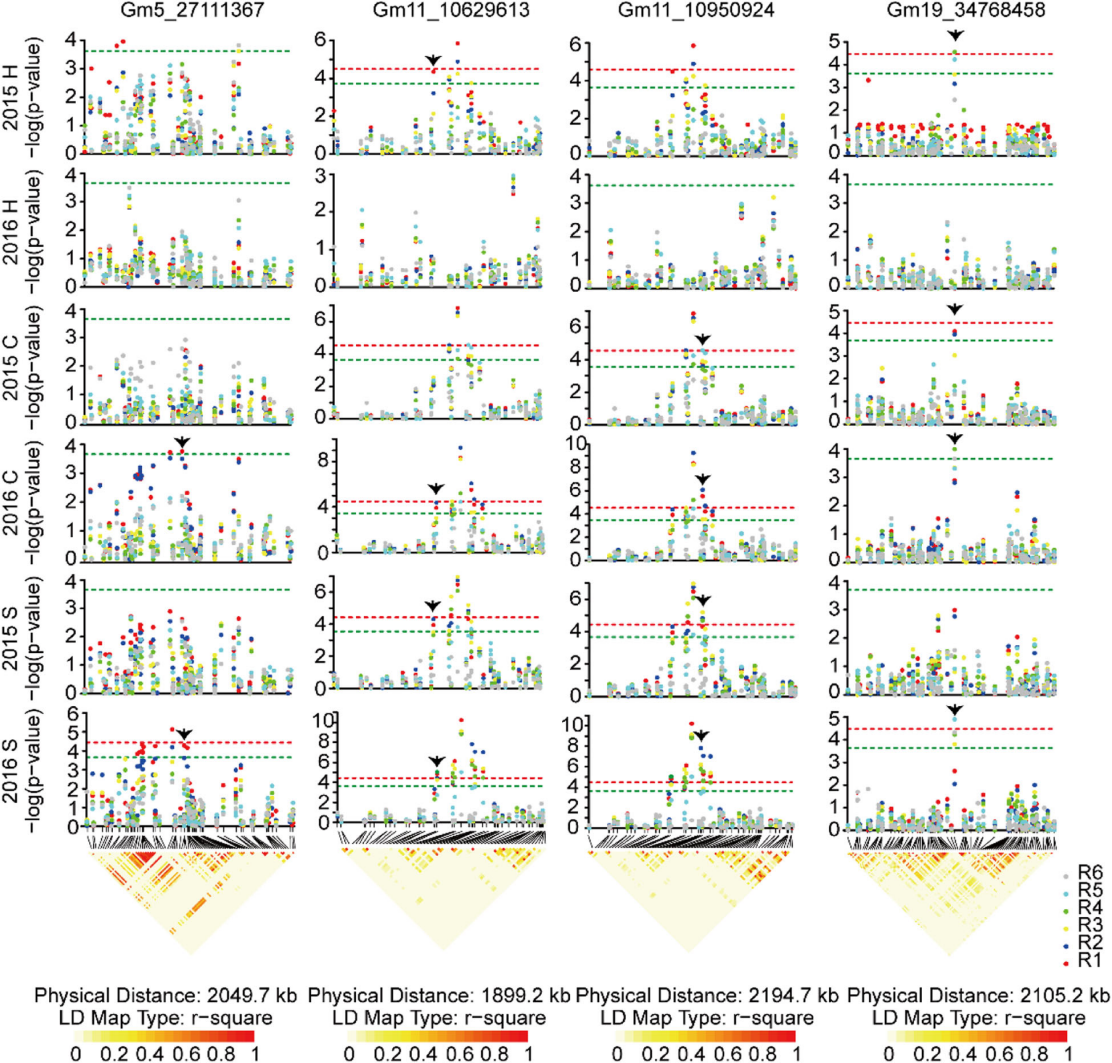
Manhattan plot and LD block of Gm5_27111367 (Gm5_26143758~28,193,474), Gm11_10629613 (Gm11_9712686~11,611,890), Gm11_10950924 (Gm11_9745828~11,940,522) and Gm19_34768458 (Gm19_33680089~35,785,309). Black arrow indicated target SNPs. The up panel was the Manhattan plots of negative log_10_-transformed *P*-values vs. SNPs, the significant (−log_10_*P* > 3.75) or extremely significant (−log_10_*P* > 4.44) threshold was denoted by the green or red line. The down panel was haplotype block based on pairwise linkage disequilibrium r^2^ values. R1: Flowering time; R2: Full bloom; R3: Beginning pod; R4: Full pod; R5: Beginning seed; R6: Full seed. 2015 H: 2015 Harbin; 2016 H: 2016 Harbin; 2015 C: 2015 Changchun; 2016 C: 2016 Changchun; 2015 S: 2015 Shenyang; 2016 S: 2016 Shenyang

**Fig. 6:**
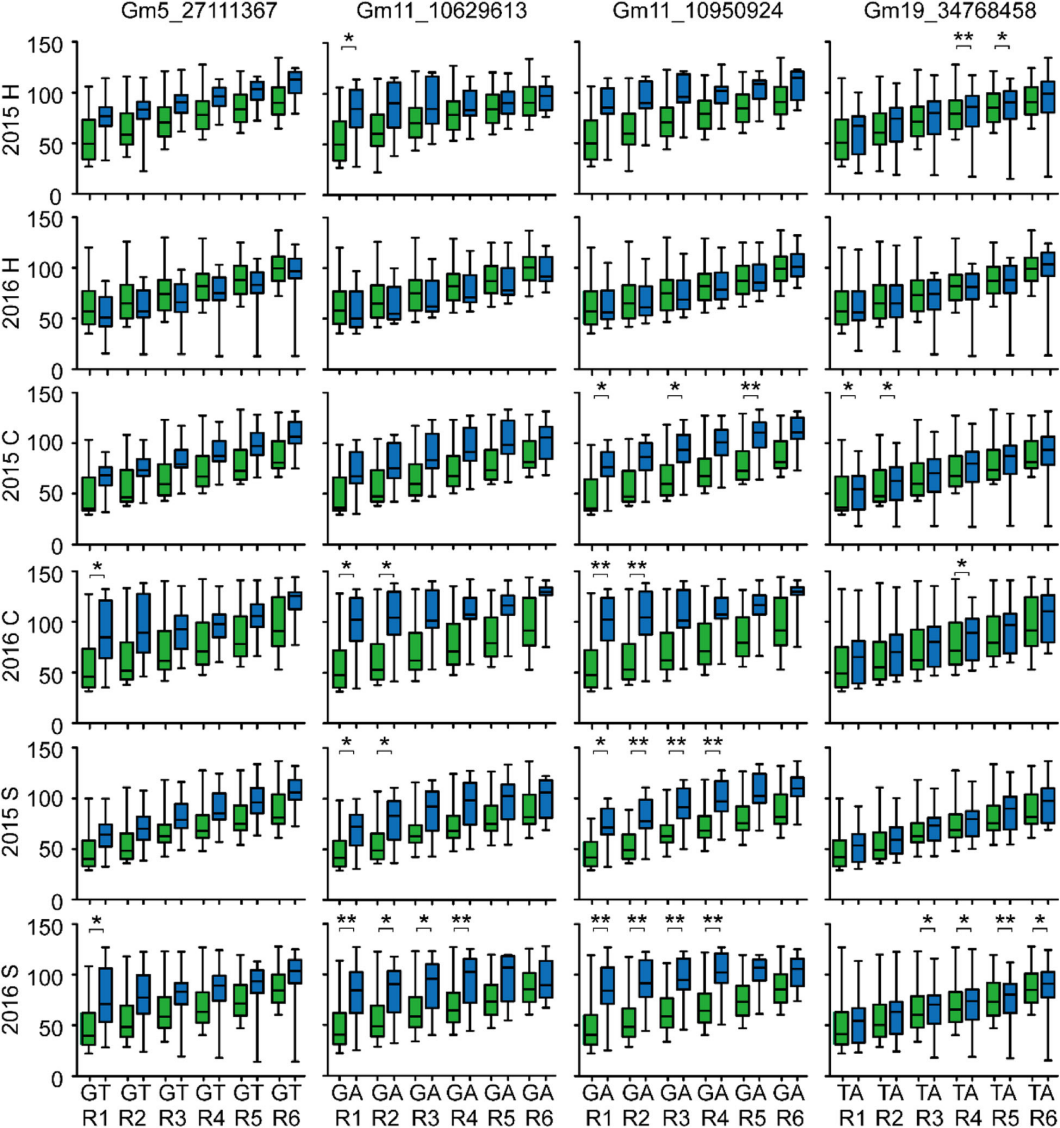
Phenotypic statistics for soybeans carrying two alleles of four SNPs in six environments. The box plot showed the differences in flowering time of the varieties carrying two alleles of different SNPs, the major and minor alleles of significant loci was marked by green and blue, respectively. R1: Flowering time; R2: Full bloom; R3: Beginning pod; R4: Full pod; R5: Beginning seed; R6: Full seed. 2015 H: 2015 Harbin; 2016 H: 2016 Harbin; 2015 C: 2015 Changchun; 2016 C: 2016 Changchun; 2015 S: 2015 Shenyang; 2016 S: 2016 Shenyang. ^*^ and ^**^ indicated that the threshold value -log_10_*P* were greater than 3.75 and 4.44, respectively

Twenty-three of 37 SNPs were located within the known QTLs or located 75 kb near the known SNPs controlling soybean growth periods, indicating the feasibility of the natural population for GWAS (Additional file 8). In addition, 14 unreported SNPs (Gm1_1929268, Gm1_55250122, Gm2_12136054, Gm2_12243533, Gm3_15104432, Gm3_45621167, Gm5_27111367, Gm9_49099305, Gm12_6106377, Gm14_3236959, Gm15_46580578, Gm17_32842602, Gm19_715196, Gm19_34768458) that may control soybean flowering were found on ten chromosomes 1, 2, 3, 5, 9, 12, 14, 15, 17 and 19. A total of 291 genes (Additional file 9) within the linkage disequilibrium (LD) block (r^2^ > 0.5) of 37 significant SNPs were screened, and we further predicted five homologs (*Glyma.05G101800*, *Glyma.11G140100*, *Glyma.11G142900*, *Glyma.19G099700*, *Glyma.19G100900*) (Table 1) of flowering time genes of *AT5G48385.1*, *AT3G46510.1*, *AT5G59780.3*, *AT1G28050.1*, and *AT3G26790.1* in Arabidopsis that played important roles in flowering pathway as candidate genes related to soybean flowering time within the 90 kb genomic region of four significant SNPs (Gm5_27111367, Gm11_10629613, Gm11_10950924, Gm19_34768458) (Fig. 5). *Glyma.05G101800* encoding FRIGIDA-like protein was located at 47.91 kb upstream of Gm5_27111367, and 251 soybeans with major allele G at this locus flowered 23.82, 19.33, 34.94, 19.03, and 32.07 days earlier than the 27 soybeans with minor allele T in five environments of 2015 Harbin, 2015 Changchun, 2016 Changchun, 2015 Shenyang, 2016 Shenyang, respectively (Fig. 6). *Glyma.11G140100* encoding PUB13 (plant U-box 13) protein was located at 47.56 kb downstream of Gm11_10629613, and 253 soybeans carrying major allele G at this locus flowered 28.23, 22.01, 37.48, 22.72, and 33.90 days earlier than the 25 soybeans with minor allele A in 2015 Harbin, 2015 Changchun, 2016 Changchun, 2015 Shenyang, 2016 Shenyang, respectively (Fig. 6). *Glyma.11G142900* encoding MYB59 protein was located at 35.11 kb upstream of Gm11_10950924, and 251 soybeans with major allele G at this locus flowered 33.51, 29.13, 44.52, 26.27, and 39.73 days earlier than the 27 soybeans with minor allele A in 2015 Harbin, 2015 Changchun, 2016 Changchun, 2015 Shenyang, 2016 Shenyang, respectively (Fig. 6). *Glyma.19G099700* and *Glyma.19G100900* encoding CONSTANS and FUS3 proteins were located at 85.90 and 37.60 kb downstream of Gm19_34768458, respectively, and 238 soybeans with the major frequency allele T at this locus flowered 7.68, 9.21, 5.72, 6.10, and 7.56 days earlier than the 40 soybeans with the alternative allele A in 2015 Harbin, 2015 Changchun, 2016 Changchun, 2015 Shenyang, 2016 Shenyang, respectively (Fig. 6). The other growth periods also showed the similar tendency with the first flowering time between two alleles of each associated SNP marker (Fig. 6). These four markers Gm5_27111367, Gm11_10629613, Gm11_10950924, and Gm19_34768458 could be targets for breeders for marker assisted selection of soybean growth periods traits.

**Table 1:** Five candidate genes related to soybean flowering time

Candidate Genes	Locus	Annotation	Distance from a gene to SNP (kb)	Functional description
Glyma.05G101800	Gm5_27111367	AT5G48385.1	−47.91	FRIGIDA-like protein
Glyma.11G140100	Gm11_10629613	AT3G46510.1	+ 47.56	plant U-box 13
Glyma.11G142900	Gm11_10950924	AT5G59780.3	−35.11	Transcription factor *MYB59*-related
Glyma.19G099700	Gm19_34768458	AT1G28050.1	−85.90	Zinc finger protein CONSTANS-LIKE 14-related transcription factor
Glyma.19G100900	Gm19_34768458	AT3G26790.1	+ 37.60	B3 domain-containing transcription factor *FUS3*

If the candidate gene is located upstream of the SNP, the distance from the gene to the SNP is indicated by a negative sign. Instead, it is represented by a positive sign

## Discussion

### Six soybean growth periods were significantly affected by genetic-environment interaction

Soybean is a short-day plant with induced cumulative effects by short days, and the flowering time of soybeans and other growth periods were quantitative traits controlled by multiple genes. The six growth periods (flowering time, full bloom, beginning pod, full pod, beginning seed, and full seed) of 278 soybean germplasm resources in this study were highly variable (14.9~43.6%) in different environments, indicating that the natural population could be used for the genetic improvement of growth periods. The high heritability (94.7~96.2%) of six growth periods indicated that they were mainly affected by genetic factors. In addition, soybean growth periods were significantly or extremely significantly affected by environmental and genotype-environment interaction, indicating that in addition to genetic effects, photoperiod and temperature conditions in different planting environments played crucial roles in determining the growth periods, which directly determined whether soybeans grown in different ecological environments could flower and mature normally. The growth periods of soybean determined the latitude range suitable for planting, so it was of great significance to study the characteristics of soybean growth periods. In this study, the genetic relationship among 94 stable soybean germplasms, including 41 earlier and 53 later flowering soybean varieties screened by GGE was far from each other, which could be qualified as hybrid breeding parent [36].

### The LD decay rate of soybean was higher than cross-pollinated species due to genetic bottleneck

Increased LD was a hallmark of genetic bottlenecks, the greater LD decay rate for self-pollination was expected to be higher than that of cross-pollinated species [37]. As the physical distance increases, the LD decay of the entire genome was estimated to be decayed to r^2^ = 0.5 within approximately 300 kb, consistent with previous studies in soybean (250~375 kb) [34], similar to the other self-pollinated species such as rice (123~167 kb) and sorghum (150 kb) [38, 39], but much larger than the cross-pollinated species such as maize (1~10 kb) [40]. The lower density of SNPs was suitable for GWAS in soybean as compared with other crops like rice, sorghum and maize, therefore, LD decay rate was the primary factor limiting the mapping resolution in GWAS for soybean.

### Determination of 23 known and 14 new soybean flowering time loci

To date, a number of QTLs associated with soybean growth periods had been reported. In the present study, a total of 37 SNPs distributed on ten chromosomes (chromosomes 1, 2, 3, 5, 9, 12, 14, 15, 17 and 19) were associated with soybean flowering time or the other growth periods in two or more environments. Among the 37 environmental stable association signals, 23 SNPs were overlapped with known QTL or located 75 kb near the known SNPs controlling soybean growth periods. For instance, two SNPs, Gm2_12243099 and Gm3_5483526, were identified at 73.01 and 18.97 kb near Gm2_12316110 [28] and Gm03_5502496 [27], respectively. All the four SNPs, Gm11_10774464, Gm11_10774489, Gm11_10793174, and Gm11_10851924 were identified near Gm11_10847172 [28]. In addition, 14 new SNPs were identified to be significantly different from the major QTLs or reported SNPs and the molecular mechanisms of these new loci needed to be further studied.

### Five candidate genes were identified in different flowering pathways

The regulation of flowering time is a very complicated process that is influenced by both genetic factors and external environmental factors. The precise control of flowering time is achieved by the combination of various signals generated by these two aspects [41]. To date, approximately six genetic pathways for the promotion or repression of flowering time have been identified in Arabidopsis, including photoperiod, temperature, vernalization, gibberellin (GA) biosynthesis, autonomous and aging pathways [42]. In photoperiod pathway, *Arabidopsis thaliana* is a genetic model system for photoperiodic responses in plants, and flowers earlier in long days than in short days. In the present study, five homologous genes (*Glyma.05G101800*, *Glyma.11G140100*, *Glyma.11G142900*, *Glyma.19G099700*, *Glyma.19G100900*) of Arabidopsis flowering genes of *AT5G48385.1*, *AT3G46510.1*, *AT5G59780.3*, *AT1G28050.1*, and *AT3G26790.1* that played important roles in Arabidopsis flowering pathway were identified to probably participate in the regulation of soybean growth periods based on strong correlation peak SNP and LD block of four significant SNPs (Gm5_27111367, Gm11_10629613, Gm11_10950924, Gm19_34768458). The Arabidopsis transcription factor *CONSTANS (CO)* played a central role in promoting flowering in LDs. CO protein directly binded to the motifs in the proximal promoter of its major target gene *FLOWERING LOCUS T (FT)* to promote flowering [43, 44]. After transcriptional activation by *CO*, FT protein moved to the shoot apex, where it induced transcriptional reprogramming of the meristem to form an inflorescence meristem and subsequently flowered [45]. Thus, *Glyma.19G099700*, located at 85.90 kb downstream of Gm19_34768458, and encoding a *ZINC FINGER PROTEIN CONSTANS-LIKE 14-RELATED* transcription factor might be involved in soybean photoperiod control of flowering pathway. In vernalization pathway, *FRI* (*FRIGIDA*) was located upstream of *FLC* (*FLOWERING LOCUS C*) and regulated vernalization by regulating *FLC* [42]. The two helix-helical domains of FRI protein interacted with the nucleus cap-binding complex (CBC) to increase transcription and efficient splicing of the flowering inhibitor *FLC* and delayed flowering [46]. Then, a FRIGIDA-like protein encoded by *Glyma.05G101800* was located at 47.91 kb upstream of Gm5_27111367, but there was no vernalization in soybean, and the function of soybean FRI needed to be validated whether it was related to flowering time. PUB13 was a negative regulator of flowering time under middle- and long-day conditions in which the expression of SOC1 (SUPPRESSOR OF OVEREXPRESSION OF CONSTANS1) and *FT* was induced while *FLC* expression was suppressed in Arabidopsis [47]. *Glyma.11G140100* located at 47.56 kb upstream of Gm11_10629613 might be considered to be associated with soybean flowering. *MYB59* was induced during the light-to-dark transition [48] and was regulated by the circadian cycle with peak expression in the evening, probably due to its regulation by *CIRCADIAN CLOCK ASSOCIATED 1 (CCA1)* which was expressed in the morning [49]. CCA1 acted as a transcriptional repressor by associating to the *ELF4* promoter [50] and the *ELF3* promoter [51] of the photoperiodic flowering pathway. Therefore, the transcription factor *MYB59*-related *Glyma.11G142900* located at 35.11 kb upstream of Gm11_10950924 might be considered to be associated with soybean flowering. *Glyma.19G100900* located at 37.60 kb downstream of Gm19_34768458 was a B3 domain-containing transcription factor *FUS3*, which was also identified as a candidate gene of soybean flowering time. Arabidopsis plants overexpressing *FUS3* post-embryonically in the L1 layer (*ML1p:FUS3*) showed late flowering and other developmental phenotypes [52].

## Conclusions

GWAS was powerful in dissecting complex traits and identifying candidate genes. Fourteen novel SNPs and 23 SNPs that located within known QTLs or 75 kb near the known SNPs associated with soybean flowering time or other related growth period traits in this study may have great potential for soybean yield improvement. Five candidate genes related to soybean flowering time might serve as promising targets for studies of molecular mechanisms underlying growth period traits in soybean.

## Methods

### Plant materials and phenotypic evaluation

The soybean growth period traits including flowering time, full bloom, beginning pod, full pod, beginning seed, and full seed of the natural population consisting of 278 diverse soybean accessions with varied maturity and growth habit characteristics were recorded and used for GWAS. Two hundred sixty-four and fourteen soybean germplasms [53] were from China and other countries (20°13′N~ 61.5°N) (Fig. 1, Additional file 1). The map was completed by ‘ggplots’, ‘colorspace’, ‘ggmap’, ‘sp’, ‘maptools’, ‘maps’, and ‘labeling’ packages in R software [54, 55]. All the soybean germplasms were sowed in experimental farms in three different latitudes of Harbin (45°75’N, 126°63’E), Changchun (43°88’N, 125°35’E) and Shenyang (41°44’N, 123°30’E) in China using randomized complete block designs with three replicates in 2015 and 2016. Each experimental block consisted of 2 m long rows with 0.6 m row spacing and 0.05 m plant spacing. The soybean emergence stages and the reproductive periods such as flowering time, full bloom, beginning pod, full pod, beginning seed, and full seed were recorded as described by Fehr and Caviness [56], and the days from emergence to reproductive periods were calculated. Each stage was defined to have occurred if at least 50% of the individual plants of a given soybean variety had reached that stage.

### Statistical analysis

The descriptively statistical analyses were carried out by SPSS19.0 [57]. The phenotypic data of flowering time, full bloom, beginning pod, full pod, beginning seed, and full seed for 278 soybean germplasms were ranked by individual cases and transformed into variables obeying standard normal distribution using SPSS19.0 [57] for correlation analysis, variance analysis (ANOVA) and GGE biplot. Correlation analysis of flowering time and full bloom, beginning pod, full pod, beginning seed, full seed in six environments were identified using Pearson’s correlation coefficients of “Performance Analytics” package in R software [31]. ANOVA was performed following the standard procedure of a mixed effect model by DPS v14.1.0 to determine the level of the significance of genotype differences, locations, cultivation years and their interactions [58]. Genotype and location were considered as fixed effects, while year was considered as a random effect. The phenotypic observation *Z*_*ijkr*_ was modeled as:

$$ {Z}_{ij k r}=\upmu +{G}_i+{L}_j+{Y}_k+{B}_r\left({L}_j{Y}_k\right)+{GL}_{ij}+{GY}_{ik}+{LY}_{jk}+{GL Y}_{ij k}+{e}_{ij k r} $$

where *Z*_*ijkr*_ was the response variable; μ was the grand mean; *G*_*i*_ was the genotype effect; *L*_*j*_ was the location effect; *Y*_*k*_ was the year effect; *B*_*r*_(*L*_*j*_*Y*_*k*_) was the block effect; *GL*_*ij*_ was the genotype-by-location interaction; *GY*_*ik*_ was the genotype-by-year interaction; *LY*_*jk*_ was the location-by-year interaction; *GLY*_*ijk*_ was the genotype-by-location-by year interaction and *e*_*ijkr*_ was the residual error. These components were used to calculate broad-sense heritability (*h*^*2*^) for soybean flowering time, full bloom, beginning pod, full pod, beginning seed, and full seed, the calculation is based on the following formula [59]:

$$ {h}^2=\frac{{\upsigma_g}^2}{{\upsigma_g}^2+{\upsigma_{gy}}^2/y+{\upsigma_{gl}}^2/l+{\upsigma_{gl y}}^2/ ly+{\upsigma_{\varepsilon}}^2/ rly} $$

where σ_*g*_^2^ was genotype, σ_*gl*_^2^ was genotype-by-location, σ_*gy*_^2^ was genotype-by-crop year, σ_*gly*_^2^ was genotype-by-location-by-crop year, σ_*ε*_^2^ was error, *r* was number of replications, *l* was number of locations and *y* was crop years respectively.

GGE biplot completed by “GGEBiplotGUI” package in R software [60] was used to screen the stable soybean varieties with early flowering and late flowering in multi-environment. The general model of GGE biplot based on singular value decomposition (SVD) of environment-centered or environment-standardized [61] could be written as:

$$ {Y}_{ij}-\mu -{\beta}_j={\lambda}_1{\xi}_{i1}{\eta}_{j1}+{\lambda}_2{\xi}_{i2}{\eta}_{j2}+{\varepsilon}_{ij} $$

where *Y*_*ij*_ was the measured mean of *i*^*th*^ genotype in *j*^*th*^ environment; *μ* was the grand mean; *β*_*j*_ was the main effect of *j*^*th*^ environment; *μ* + *β*_*j*_ was the average trait over all genotypes in *j*^*th*^ environment; *λ*_1_ and *λ*_2_ were the singular values for the first and second principal component (PC1 and PC2); *ξ*_*i*1_ and *ξ*_*i*2_ were eigenvectors of *i*^*th*^ genotype for PC1 and PC2; *η*_*j*1_ and *η*_*j*2_ were eigenvectors of *j*^*th*^ environment for PC1 and PC2; *ε*_*ij*_ was the residual of the model associated with *i*^*th*^ genotype in *j*^*th*^ environment.

### Genotyping and quality control

The double enzyme group comprising *Mse*I and *Hae*III (Thermo Fisher Scientific Inc., Waltham, MA, USA.) was used to digest the soybean genomic DNA that isolated from the fresh leaves of a single plant [62] into more than 50,000 sequencing tags, based on which, the sequencing libraries of each accession were constructed [33, 63]. The Short Oligonucleotide Alignment Program 2 was used to map raw paired-end reads of the 45 bp sequence read at both ends of the sequencing tags for each library, which was obtained using the barcode approach combined with the Illumina Genome Analyzer II (Illumina Inc., San Diego, CA, USA) onto the reference genome [64]. Approximately 58,000 high-quality SLAF tags were obtained after sequencing reads with the same genomic position of each accession. A total of 34,710 SNP loci with missing rate less than 10% and minor allele frequency (MAF) greater than 0.04 was used for GWAS.

### Linkage disequilibrium (LD), population structure and kinship analyses

Pairwise LD between 34,710 SNP loci with missing rate less than 10% and MAF greater than 0.04 was estimated using squared allele frequency correlations (r^2^) in TASSEL 5.0, the LD decay rate of the population was measured as the chromosomal distance when r^2^ dropped to half its maximum value [38, 65, 66]. The 34,710 SNP loci were also used to perform principal component analysis and calculate kinship matrixes by identity-by-state (IBS) method implemented in TASSEL 5.0 to infer population stratification and relatedness among individuals [65, 67].

### GWAS and candidate genes prediction

The statistical mixed linear model (CMLM-PCA + K) was used to perform GWAS using TASSEL 5.0 [65]. The equation for the CMLM-PCA + K analysis was expressed as:

$$ y=X\upalpha +P\upbeta +Z\mathrm{u}+\mathrm{e} $$

where *y* was phenotype value; α was the vector of SNP effects; β was vector of population structure effects based on PCA; u was vector of kinship background effects; e was vector of residual effects; *X*, *P*, *Z* were incidence matrix relating the individuals to fixed marker effects α, fixed principal component (PC) effects β, random group effects u, respectively.

Bonferroni test *P* < 0.05/n or *P* < 0.01/n (*n* = 278), that was -log_10_*P* > 3.75 or -log_10_*P* > 4.44, was used to determine the significant or extremely significant SNP-trait associations. The R-based package snp.plotter was used to comb the LD block of the significant SNPs, the candidate genes associated with soybean flowering time within which were predicted using the SoyBase (https://www.soybase.org) and TAIR (https://www.arabidopsis.org/) databases.

## Supplementary information

**Additional file 1.** General information and phenotypic data of 278 accessions used in this study.

**Additional file 2.** Statistical analysis of growth periods traits for 278 soybean varieties in six environments.

**Additional file 3.** Variance analysis (ANOVA) of 278 soybean varieties in six environments.

**Additional file 4.** The soybeans with stable below- or above-average flowering time.

**Additional file5.** The raw data and the corresponding accession numbers by SLAF-seq.

**Additional file 6.** SNPs distribution on each chromosome.

**Additional file 7.** Peak SNP associated with soybean growth periods in one environment by CMLM-PCA + K model.

**Additional file 8.** Peak SNPs associated with soybean growth periods in at least two environments by CMLM-PCA + K model.

**Additional file 9.** The 291 genes within the LD block (r^2^ > 0.5) of 37 significant SNPs.

**Additional file 10: Figure S1.** The normal score of standard normal random variable transformed from growth periods for 278 soybeans.

**Additional file 11: Figure S2.** Phenotypic correlation analysis of 278 soybean varieties in six environments.

**Additional file 12: Figure S3.** Manhattan and QQ plots of GWAS for soybean growth periods.

## Data Availability

All data generated or analyzed during this study are included in this published article and its supplementary information files.

## Abbreviations

*CMLM*: Compressed mixed linear model
*GWAS*: Genome-wide association study
*LD*: Linkage disequilibrium
*MAF*: Minor allele frequencies
*PCA*: Principal component analysis
*R*^*2*^: Phenotypic variance
*SLAF-seq*: Specific-locus amplified fragment sequencing
*SNP*: Single nucleotide polymorphisms
