# Supplementary material for: Identification of genetic loci and candidate genes related to soybean flowering through genome wide association study
Source: BMC Genomics. 2019 Dec 16;20:987. doi: 10.1186/s12864-019-6324-7 (PMC6916438; doi:10.1186/s12864-019-6324-7)

**Fig. S1 The normal score of standard normal random variable transformed from growth periods for 278 soybeans.**

The abscissa axis represented the normal score, and the ordinate axis represented the frequency. R1: Flowering time; R2: Full bloom; R3: Beginning pod; R4: Full pod; R5: Beginning seed; R6: Full seed. 2015 H: 2015 Harbin; 2016 H: 2016 Harbin; 2015 C: 2015 Changchun; 2016 C: 2016 Changchun; 2015 S: 2015 Shenyang; 2016 S: 2016 Shenyang.


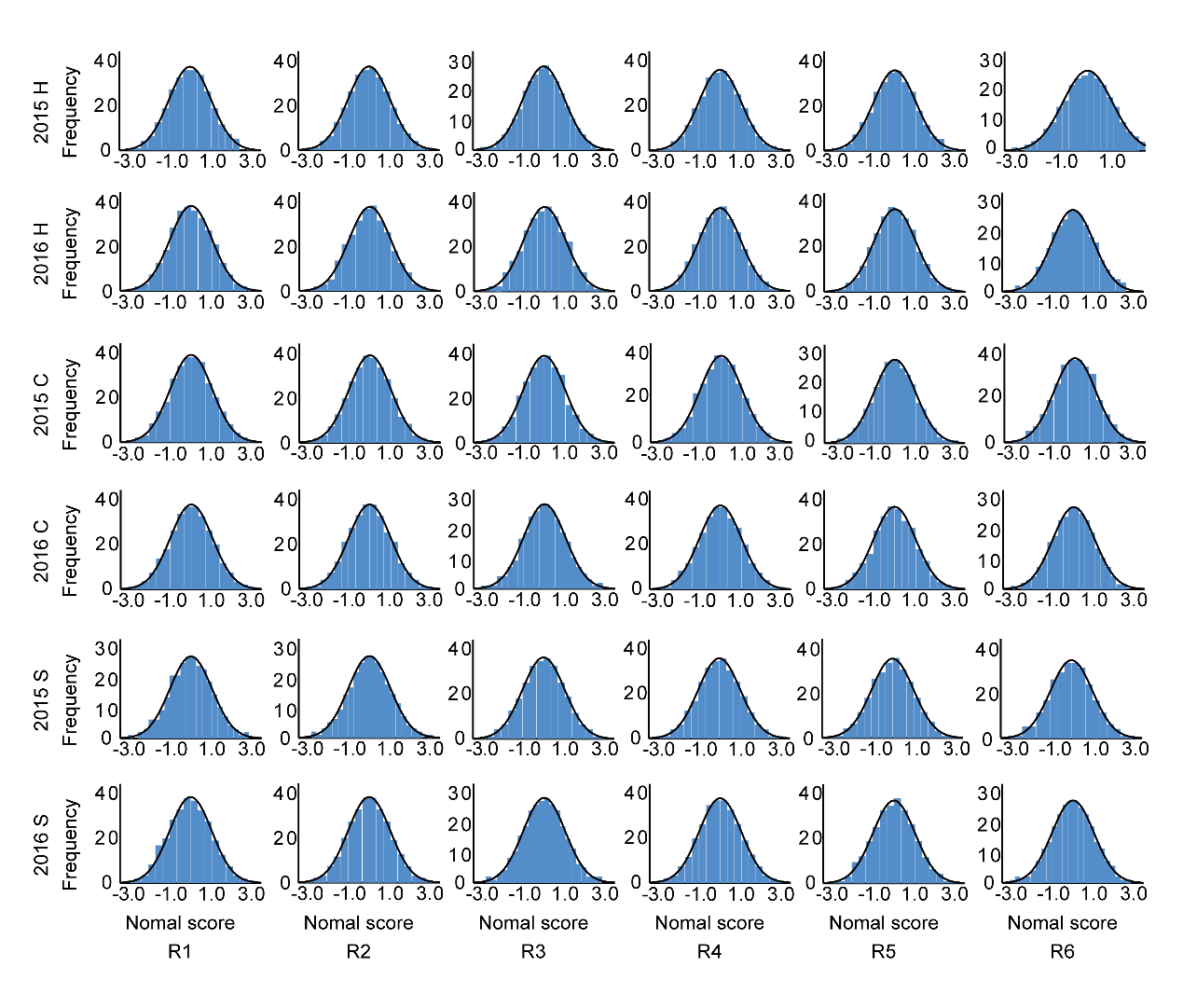

Supplement: Supplementary file 10 — Additional file 10: Figure S1. The normal score of standard normal random variable transformed from growth periods for 278 soybeans. [file 12864_2019_6324_MOESM10_ESM.docx]
