# Supplementary material for: Identification of genetic loci and candidate genes related to soybean flowering through genome wide association study
Source: BMC Genomics. 2019 Dec 16;20:987. doi: 10.1186/s12864-019-6324-7 (PMC6916438; doi:10.1186/s12864-019-6324-7)

**Fig. S2 Phenotypic correlation analysis of 278 soybean varieties in six environments.**

(a) 2015 Harbin. (b) 2015 Changchun. (c) 2015 Shenyang. (d) 2016 Harbin. (e) 2016 Changchun. (f) 2016 Shenyang. ^***^: *P*<0.001, ^**^: *P*<0.01, ^*^: *P*<0.05.

R1: Flowering time; R2: Full bloom; R3: Beginning pod; R4: Full pod; R5: Beginning seed; R6: Full seed.


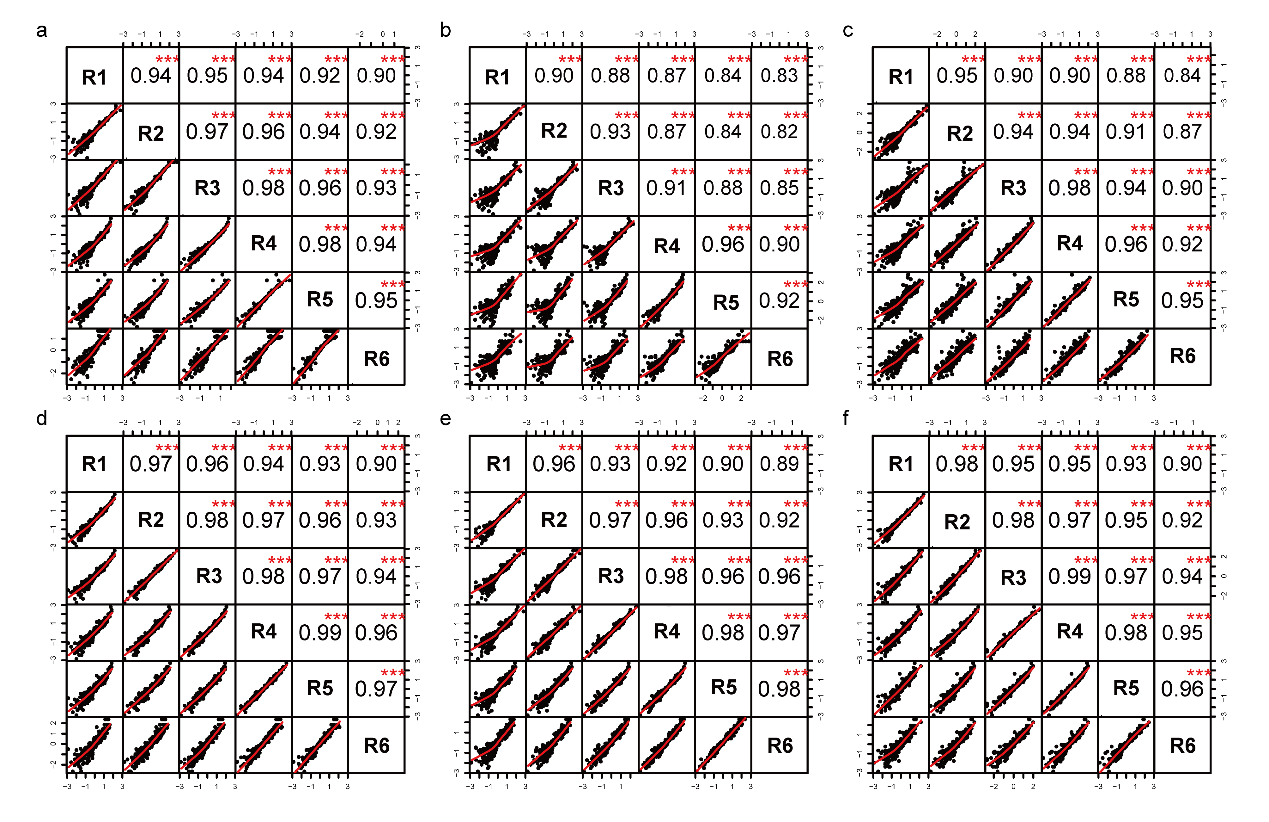

Supplement: Supplementary file 11 — Additional file 11: Figure S2. Phenotypic correlation analysis of 278 soybean varieties in six environments. [file 12864_2019_6324_MOESM11_ESM.docx]
