# Supplementary material for: Identification of genetic loci and candidate genes related to soybean flowering through genome wide association study
Source: BMC Genomics. 2019 Dec 16;20:987. doi: 10.1186/s12864-019-6324-7 (PMC6916438; doi:10.1186/s12864-019-6324-7)

**Fig. S3. Manhattan and QQ plots of GWAS for soybean growth periods.**

The ordinate axis represented the negative log_10_-transformed P values of SNPs from a genome-wide scan. The abscissa axis represented the chromosome. The significant trait-associated SNPs were distinguished by the threshold (-log_10_*P*>3.75) line which colored in blue.

R1: Flowering time; R2: Full bloom; R3: Beginning pod; R4: Full pod; R5: Beginning seed; R6: Full seed.


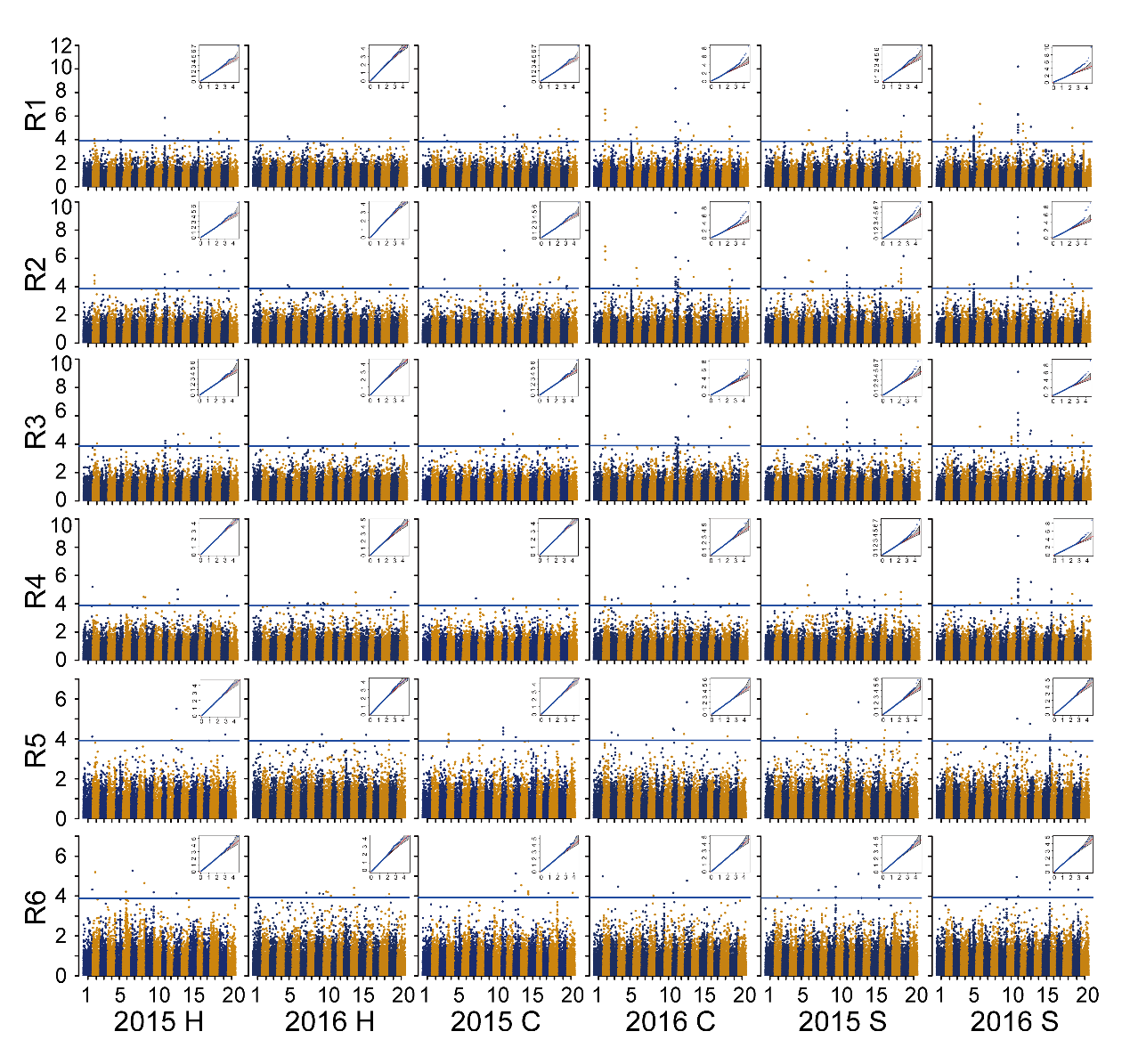

Supplement: Supplementary file 12 — Additional file 12: Figure S3. Manhattan and QQ plots of GWAS for soybean growth periods. [file 12864_2019_6324_MOESM12_ESM.docx]
